# Intravenous Methylprednisolone Pulse Therapy Versus Intravenous Immunoglobulin in the Prevention of Coronary Artery Disease in Children with Kawasaki Disease: A Randomized Controlled Trial

**DOI:** 10.7759/cureus.26252

**Published:** 2022-06-23

**Authors:** Nahid Aslani, Seyed-Reza Raeeskarami, Ehsan Aghaei-Moghadam, Fatemeh Tahghighi, Raheleh Assari, Payman Sadeghi, Vahid Ziaee

**Affiliations:** 1 Department of Pediatrics, Children’s Medical Center, Pediatrics Center of Excellence, Tehran, IRN; 2 Department of Pediatrics, Isfahan University of Medical Sciences, Tehran, IRN; 3 Department of Pediatrics, Tehran University of Medical Sciences, Tehran, IRN; 4 Department of Pediatrics, Pediatric Rheumatology Society of Iran, Tehran, IRN; 5 Department of Pediatrics, Pediatric Rheumatology Research Group, Rheumatology Research Center, Tehran University of Medical Sciences, Tehran, IRN

**Keywords:** systemic steroid therapy, pulse steroid therapy, intravenous immunoglobulin (ivig), intravenous methylprednisolone pulse, coronary artery lesion (cal), kawasaki disease (kd)

## Abstract

Background: Kawasaki disease (KD) is often complicated by coronary artery lesion (CAL), including dilatation or aneurysms. Intravenous immunoglobulin (IVIG) is used with aspirin to prevent CAL in KD.

Objective: Given that the primary treatment for other vasculitis is the use of corticosteroids, this study has been performed to evaluate the effect of intravenous methylprednisolone pulse (IVMP) therapy in preventing CAL in KD.

Method: A randomized, single-blind clinical trial was conducted on 40 KD patients aged six months to five years. Patients were randomized into two groups according to the main treatment plan in addition to aspirin: case group (IVMP for three consecutive days and then oral prednisolone for three days) and control group (intravenous immunoglobulin 2 g/kg). Echocardiography was performed for all children at least three times, during the acute phase, two weeks, and two months later.

Results: Data analysis at the end of the study was done on 40 patients (20 patients in each group). There were no significant differences in age and sex distribution, mean fever, and acute phase duration, as well as baseline echocardiography in the two groups. The frequency of CAL was 20% in the case group and 45% in the control group, after two weeks (p<0.05), but there was no significant difference between two groups in types of coronary artery lesion after two weeks and the frequency and severity of CAL after two months.

Conclusion: IVMP as initial line therapy effectively control systemic and vascular inflammation and decrease coronary artery damage in KD.

## Introduction

Kawasaki disease (KD) is an acute, self-limited medium vessel vasculitis most commonly affecting infants and children <five years of age [1,2]. Complete KD requires persistent fever ≥five days plus four out of five clinical criteria, including bilateral non-exudative bulbar conjunctivitis, polymorphous non-vesicular rash, oropharyngeal changes, unilateral cervical lymphadenopathy, and swelling of extremities followed by desquamation [2]. Patients with prolonged fever and less than four clinical criteria have incomplete KD, and the diagnosis is based on laboratory findings or echocardiography.

Coronary artery aneurysm is a well-recognized complication of KD, occurring in roughly 20% of untreated diseases, and is the leading cause of acquired heart disease in developed countries [3,4]. Two classification criteria exist for the diagnosis of coronary artery dilation and aneurysms. The Japanese Ministry of Health (JHM) criteria classify coronary arteries using absolute or relative internal lumen diameter [5]. Dilation is defined as an internal lumen diameter >3 mm in children of age <five years, or >4 mm in children of age ≥five years, or if the internal diameter of a segment measures ≥1.5 times that of an adjacent segment. JMH criteria are more commonly used in Japan, and given the variability in lumen sizes concerning body size, it may underestimate the incidence of coronary artery dilations and aneurysms. The 2004 American Heart Association (AHA) adjusts for body surface area and classifies solely on Z-scores. Per AHA criteria, dilation is defined as a Z-score ≥2 and <2.5, and aneurysms are diagnosed if Z-score is ≥2.5. Dilation often resolves within four to eight weeks after fever onset. Giant aneurysms, defined as ≥8 mm per JHM and AHA or Z-score ≥10 per AHA criteria, are unlikely to regress [6].

Intravenous immunoglobulin (IVIG) administered in the acute stage of KD rapidly eliminates inflammation symptoms and decreases the frequency of coronary artery lesions (CAL) to approximately 5% [7,8]. Nearly 35 years later, according to the latest guidelines of the AHA in 2017, a single high dose of IVIG together with acetylsalicylic acid was still the standard recommended treatment for KD [6].

IVIG, also known as gamma globulin, is a therapeutic preparation comprising pooled blood donated from many healthy people. Although many clinical trials have demonstrated that immunoglobulin is effective and well-tolerated, various adverse effects have been reported. Most of these events, such as flushing, headache, malaise, fever, chills, fatigue, and lethargy, are transient and mild. However, some rare side effects are severe, including renal impairment, thrombosis, arrhythmia, aseptic meningitis, hemolytic anemia, and transfusion-related acute lung injury. These adverse effects are associated with specific immunoglobulin preparations and individual differences [8]. Approximately 5-35% of patients fail to respond to IVIG and remain febrile ≥36 hours after the IVIG infusion and are thus classified as IVIG-refractory or IVIG-resistant [9].

Corticosteroids are an effective treatment due to their well-known and potent anti-inflammatory properties in other vasculitis disorders. Their potential use in KD was considered soon after the disease was recognized and expected to benefit. Corticosteroids normalize multiple transcriptional patterns and inhibit the synthesis of the most known type 2 and proinflammatory cytokines [10]. According to the AHA scientific statement 2017, intravenous methylprednisolone (IVMP), 30 mg/kg for three days with or without an oral glucocorticoid taper, is one of the three most effective second-line therapies recommended for these patients [6].

Potential benefits of corticosteroids as rescue therapy in KD also have been reported. Corticosteroids in KD have been studied both as primary therapy and “rescue” therapy, and doses have ranged from pulse doses of 30 mg/kg (maximum of 1 g) to conventional anti-inflammatory doses (2 mg/kg/day). However, a few clinical studies have been conducted to evaluate the role of corticosteroids alone compared to IVIG. There have been many disagreements on the treatment, and none of the therapies tried thus far have proven to be sufficiently effective [11]. Although IVIG has been considered a standard treatment for KD, it is expensive and unavailable in all countries.

On the other hand, corticosteroids are the primary and effective treatment in other forms of vasculitis and one of the effective treatments in resistance KD. So, this study was conducted to investigate how initial methylprednisolone pulse treatment alone in the acute phase of KD can effectively control systemic and vascular inflammation in the acute phase of KD. In fact, in this study, we are looking to see if corticosteroids can be considered an alternative treatment for KD when IVIG is unavailable.

This article was previously published as a preprint: Aslani N, Raeeskarami SR, Aghaei-Moghadam E, Tahghighi F, Assari R, Sadeghi P, Ziaee V: Intravenous methylprednisolone pulse therapy versus intravenous immunoglobulin (IVIg) In the prevention of coronary artery disease in children with Kawasaki disease: a randomized controlled trial. Research Square. 2021. DOI: 10.21203/rs.3.rs-460428/v1

## Materials and methods

A randomized, single-blind clinical trial, registered in the Iranian Registry of Clinical Trials (ClinicalTrials.IRCT Identifier: IRCT20181202041817N1), was performed from October 2019 until October 2020 in the pediatric rheumatology division of Children's Medical Center, a tertiary pediatric referral hospital, Tehran, Iran. The Ethics committee of the university approved the study. Data collection was completed before the coronavirus disease 2019 (COVID-19) pandemic. All the patients were visited periodically after hospitalization and followed up by phone between outpatient visits.

Study groups and patients

The inclusion criteria were age group between six months to five years, symptoms in favor of complete and incomplete KD according to the AHA guideline of 2017 [6]. Patients were excluded who had atypical KD, macrophage activation syndrome, recurrent or previous history of KD, previously confirmed coronary artery lesion, congestive heart failure, chronic kidney disease, sensitivity to methylprednisolone or prednisolone, active zoster virus infection, or exposure to varicella for the last 21 days, intravenous or intramuscular usage of corticosteroids for more than three days in the last seven days, history of severe reactions to any human globulin product and registration in another study and patients who did not have completed series of three echocardiograms. The diagnosis of KD was based on the AHA criteria for complete and incomplete KD [6].

Main outcome variable

Patients were observed over two months and underwent two-dimensional echocardiography periodically. The first echocardiography was performed on the first day of KD diagnosis in the acute phase of KD. The second and third echocardiography was done after two weeks (in the sub-acute phase of KD) and two months (in the convalescent phase of KD), respectively. A pediatric cardiologist evaluated the echocardiography for all patients wholly blinded to the method and treatment outcome by the Samsung HS70 device (Seoul, South Korea: Medison Co., Ltd.) 8-12 and 4-8 probes sizes. Coronary artery dimension average was measured in the short-axis view and diastolic phase of at least three consecutive cardiac cycles. Echocardiographic views of coronary arteries included the right coronary artery (RCA), left main coronary artery (LMCA), left circumflex (LCX) proximal segment, and left anterior descending (LAD) proximal, middle segment. Subsequent echocardiography was performed at least two more times, two weeks and two months later, based on coronary artery involvement and the cardiologist's discretion.

The coronary artery lesions were classified further as ectasia (diffuse dilatation), small aneurysm (local dilatation of internal lumen diameter less than 4 mm or ≥1.5 times to the adjacent segment in children older than five years), medium aneurysm (local dilatation of internal lumen diameter >4 mm but less than 8 mm or internal lumen diameter more than 1.5 to four times to the adjacent segment in children older than five years), and large or giant aneurysm (local dilatation of internal lumen diameter >8 mm or >four times to the adjacent segment in children older than five years) based on the Japanese Ministry of Health (JHM) criteria [5].

Procedures

Our randomization method in this study used a sealed envelope system. After gaining informed consent from the parents of the patients who met the inclusion criteria and assigning the treatment randomly to the sealed envelopes, an envelope was randomly removed and opened by the participant. Then the assigned treatment regimen was selected.

**Figure 1:**
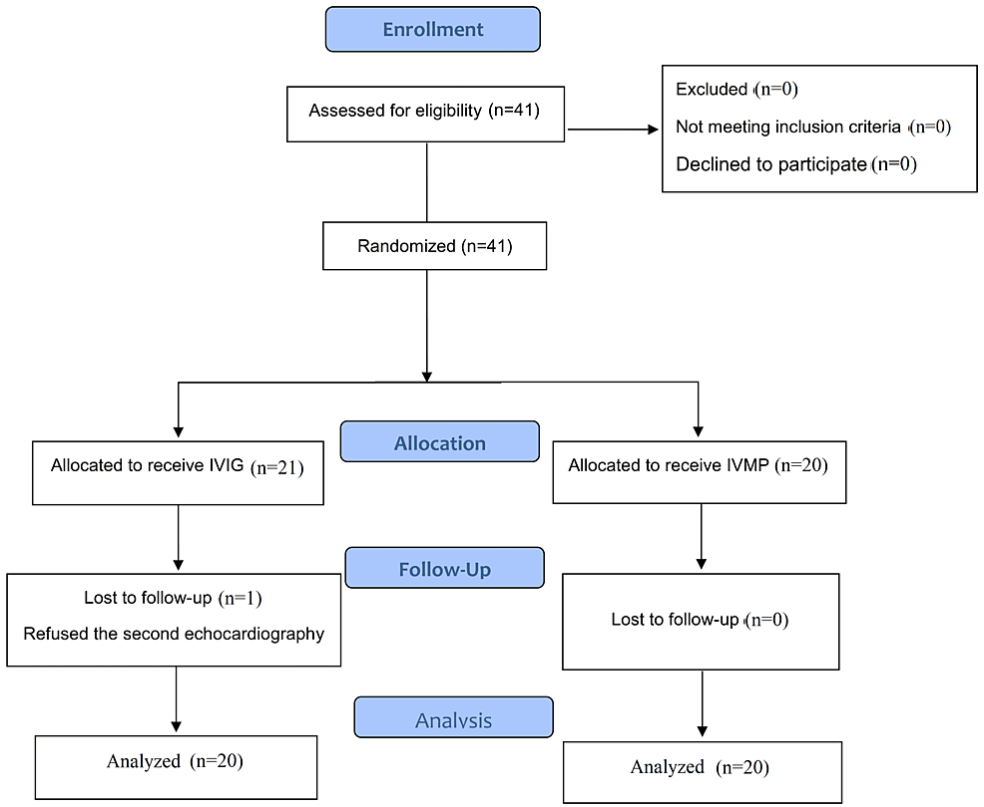
CONSORT flow chart of the randomized clinical trial with intravenous methylprednisolone pulse administration in children with KD. IVIG: intravenous immunoglobulin; IVMP: intravenous methylprednisolone pulse; CONSORT: Consolidated Standards of Reporting Trials; KD: Kawasaki disease

Then, the patients, according to the main treatment in addition to aspirin, were randomly allocated into either IVMP group (intravenous methylprednisolone pulse, 30 mg/kg/day for three consecutive days, and then oral prednisolone 1 mg/kg/day for three days group) or IVIG group (intravenous immunoglobulin 2 g/kg) in a single-blinded approach, Consolidated Standards of Reporting Trials (CONSORT) flow diagram (Figure 1). Once the patient's parents signed the informed consent, the physician administered an intravenous methylprednisolone pulse to each new patient based on the randomization protocol. The definition of response to treatment in both groups was based on the cessation of fever 12 hours after the end of IVIG infusion or the first dose of steroid pulse and reduction of c-reactive protein (CRP) 12 hours after the end of each treatment to less than 50%. The first dose of steroid pulse was considered for evaluating response to treatment. Patients who did not meet both of the treatment response criteria were considered a treatment failure, excluded from the study, and received standard treatment (second dose of IVIG 2 g/kg in the IVIG group and IVIG 2 g/kg in the IVMP group). We considered 12 hours as the response time to treatment because any patients in the IVMP group who did not respond to IV methylprednisolone pulse treatment could be excluded from the study sooner and treated with standard IVIG treatment.

Demographic, clinical, and laboratory findings of the participants were recorded, including age, sex, erythrocyte sedimentation rate (ESR), CRP, cell blood count with differential count (CBC diff), serum glutamic-oxaloacetic transaminase (SGOT), serum glutamic-pyruvic transaminase (SGPT), urinalysis, urine culture, echocardiography results, and early-onset complications.

Statistical analysis

The sample size was calculated using the Altman nomogram with a power of 80%, and as a result, it was equal to 20 people in each group. In this formula, if α is equal to 0.05 and therefore Z 1-α/2 is equal to 1.96; and β is equal to 0.2 and therefore Z 1-β is equal to 0.85. In addition, if d=0.6 and σ=0.8 is considered; as a result, the sample size (n) is equal to 20 people in each group [12].

Data were analyzed using the SPSS software version 24 (Chicago, IL: SPSS Inc.) for Windows. The Kolmogorov-Smirnov test checked the normality of variables. Mean (SD), and median (interquartile range) were used for expressing normally and non-normally distributed continuous variables. Categorical variables were reported as percentages. The mean (SD) and median (interquartile range) of continuous variables were compared between two groups using an independent t-test. For evaluation of the prevalence between two groups, the chi-square test and for evaluation of percentage McNemar test were applied. P-values less than 0.05 were considered significant.

## Results

Demographic and clinical characteristics of the subjects

We recruited 41 participants in the study; 21 children in the IVIG group and 20 children in the IVMP group. One child in the IVIG group was excluded due to refusing to perform the second echocardiography. All of the patients met both treatment response criteria, none of them were considered treatment failures, and no participants were excluded as a treatment failure from the study. Finally, data analysis for the findings, performed at the end of the study, was done on 40 patients (20 patients in the IVMP group and 20 in the IVIG group).

Patient demographic, clinical, and laboratory characteristics were similar between groups. The mean age of randomized patients in the IVMP and IVIG groups was 2.6 years and 2.7 years, respectively (p=0.6). A total of 65% of patients in the IVIG group were girls and 55% of patients in the IVMP group were boys. The proportion of girls in the pulse group was 20% higher than in the IVIG group (p=0.2), and these differences in sex and age distribution were not significant between the two groups (Table 1). Based on the chi-square test results, there were no significant differences in the sex distribution of patients in the two groups. The age range of patients was between 10 and 54 months in the IVIG group and seven to 60 months in the IVMP group.

**Table 1:** Comparison of demographic, clinical type, and characteristics of KD patients between IVIG and IVMP groups. IVIG: intravenous immunoglobulin; IVMP: intravenous methylprednisolone pulse; KD: Kawasaki disease

Variables	IVIG group	IVMP group	p-Value
Female/male	9/11 (45:55%)	13/7 (65:35%)	0.2
Mean age	2.7 years (10-54 months)	2.6 years (7-60 months)	0.6
Complete/incomplete KD	8/12 (40/60%)	13/7 (65/35%)	0.1
Mean fever duration	9.7 days	8.9 days	0.3
Mean acute phase of the disease	6.9 hours	7 hours	0.9

The mean intervals from the onset of the fever to the start of treatment were 8.9 days in the IVMP to 9.7 days in the IVIG groups and were not significantly different (p=0.3). Also, the mean duration of the acute phase from the start of treatment to the reduction in CRP index to less than 50% of initial values was about seven hours and not significantly different between the two groups (p=0.9).

Overall, 47.5% of all patients in this study met incomplete Kawasaki disease criteria. There were seven patients (35%) in the IVMP group and 12 (60%) in the IVIG group with a fever of more than five days and two or three criteria as incomplete Kawasaki patients. However, there was no significant difference between the two groups. (p=0.1).

Cardiac involvement in the acute phase of KD

The baseline echocardiography did not significantly differ in the two groups. Coronary artery ectasia was both groups' most common type of coronary artery involvement. Only a large coronary artery aneurysm was detected in one patient in the IVIG group. Nine participants (45%) in the IVMP group and 15 (75%) in the IVIG group had coronary artery involvement. The most involved coronary arteries in both groups were LMCA, RCA, and LAD, respectively, and there was no significant difference between the two groups (LMCA, p=0.3) (RCA, p=0.5) (LAD, p=0.9). There was no significant difference in coronary abnormality ratio between the two groups before treatment (p>0.05) (Table 2). No other comorbidities existed in any patient, such as congenital heart disease and aortic valve insufficiency. Only tricuspid valve regurgitation was present in both groups in approximately the same proportion.

**Table 2:** Comparison of the coronary artery involvement and other echocardiographic abnormalities in Kawasaki disease patients in IVMP and IVIG groups. IVIG: intravenous immunoglobulin; IVMP: intravenous methylprednisolone pulse; LMCA: left main coronary artery; RCA: right coronary artery; LAD: left anterior descending

Coronary artery involvement	IVIG group total number (%)	IVMP group total number (%)	p-Value
RCA	4 (20)	2 (10)	0.4
LMCA	12 (60)	8 (40)	0.3
LAD	3 (15)	3 (15)	0.9

Cardiac involvement in the sub-acute phase of KD

After two weeks, four participants (20%) in the IVMP group and 10 participants (50%) in the IVIG group had coronary artery involvement and a reduction of coronary abnormality by 20% in the IVMP group and 25% in the IVIG group were not significantly different than before treatment in each group (IVMP group: p=0.08, IVIG group: p=0.15) (Table 3).

**Table 3:** Comparison of coronary lesions of the acute and sub-acute phase of Kawasaki disease by echocardiography in IVIG and IVMP groups. IVIG: intravenous immunoglobulin; IVMP: intravenous methylprednisolone pulse

Coronary artery lesion	IVMP (%)	IVIG (%)
Acute phase (1)	9 (45)	15 (75)
Sub-acute phase (2)	4 (20)	10 (50)
Convalescent phase (3)	1 (5)	1 (5)
P-value (between phase 1 and 2)	0.08	0.15
P-value (between phase 1 and 3)	0.003	<0.001

Cardiac involvement in the convalescent phase of KD

After two months, in each group, only one patient had a small coronary artery aneurysm, and also, the rate of improvement in coronary artery abnormalities was significantly different from before treatment in each group (IVMP group: p=0.003, IVIG group: p≤0.001) (Table 3).

Changes in cardiac involvement in different phases

Five participants (80%) in the IVMP group and five (50%) in the IVIG group showed coronary artery lesion improvement on the second echocardiography based on the chi-square test result. There was a significant relationship between coronary involvement and non-involvement two weeks after treatment with the type of treatment. The prevalence of coronary artery involvement after two weeks in the IVMP group was not the same or more than the group treated with the IVIG group; it was significantly less (p=0.047). There was no significant difference in coronary abnormality ratio between the two groups two months after treatment, too (p=0.7) (Table 4).

**Table 4:** Assessment of coronary lesions of the acute, sub-acute, and convalescent phases by echocardiography between IVMP and IVIG groups. IVIG: intravenous immunoglobulin; IVMP: intravenous methylprednisolone pulse

Echocardiography	IVIG group total number (%)	IVMP group total number (%)	p-Value
Echocardiography in the acute phase			0.053
Ectasia	14 (70)	9 (45)	
Small aneurysm	0 (0)	0 (0)	
Moderate aneurysm	0 (0)	0 (0)	
Giant aneurysm	1 (5)	0 (0)	
Total	15 (75)	9 (45)	
Echocardiography in the sub-acute phase			0.047
Ectasia	9 (45)	3 (15)	
Small aneurysm	0 (0)	0 (0)	
Moderate aneurysm	1 (5)	1 (5)	
Giant aneurysm	0 (0)	0 (0)	
Total	10 (50)	4 (20)	
Echocardiography in the convalescent phase			0.7
Ectasia	0 (0)	0 (0)	
Small aneurysm	1 (5)	1 (5)	
Moderate aneurysm	0 (0)	0 (0)	
Giant aneurysm	0 (0)	0 (0)	
Total	1 (5)	1 (5)	

During the study, it was observed that none of the patients had significant side effects or showed adverse reactions to the drug.

## Discussion

Aspirin and IVIG are the recommended initial therapy in KD [13]. KD is an immune-mediated small and medium-sized vasculitis, and similar to other vasculitis, corticosteroids should logically be effective as anti-inflammatory drugs in treating KD. The available literature on corticosteroids' efficacy in treating KD is contradictory [14-16]. However, other studies, particularly the Randomized controlled trial to Assess Immunoglobulin plus Steroid Efficacy for Kawasaki disease (RAISE) study, have provided evidence of the benefits of corticosteroids plus intravenous immunoglobulin in high-risk patients with KD [17]. This finding may be due to different modes of steroids as rescue therapy or initial treatment and the different dose of steroids in KD (prednisolone 2 mg/kg/thrice-a-day versus IV methylprednisolone 30 mg/kg once-a-day for one to three days) [18]. The most compelling evidence for steroid use in KD comes from the meta-analysis done by Wooditch et al. [14]. They performed a meta-analysis of 862 children and found a significant reduction in the incidence of coronary artery aneurysms among patients who received corticosteroids and aspirin with/without IVIG compared with aspirin alone or with IVIG. Newer studies are now testing steroid plus IVIG-aspirin combination in those not responding to the initial therapy or those likely to have a more resistant disease [16-19].

However, none of the previous studies has used the regimen, which we had to use due to pressing circumstances. Due to resource constraints, in this study, we could only use aspirin and corticosteroids, and follow-up showed significant coronary artery improvement and the presence of only one patient with a small-sized coronary aneurysm in each group.

The relationship between the systemic immune response in the acute phase of KD and subsequent coronary artery damage could be related to tumor necrosis factor-α (TNF-α) and its downstream molecules as major mediators of coronary artery injury. In this disease, innate and acquired immune systems are activated. First, the innate immune system initiates the immune process by activating neutrophils. Interleukins 1, 6, 8, TNF-α, and many other cytokines, including interferon-gamma, are activated. This process may be a reason for the high risk of poor coronary artery outcomes in KD patients with prolonged fever and older age [20].

In some previous studies and a systematic review, the use of steroids in the treatment of KD (first-line or refractory cases) has also been shown to reduce the duration of fever cessation and normalization of CRP and hospitalization [16,21-24]. Miura et al. showed faster fever resolution in the IVMP group compared with the second IVIG dose. This finding is due to changes in the inflammatory cytokine levels. The more potent suppression of monocyte chemoattractant protein-1 (MCP-1) and TNF-α levels by IVMP are noteworthy for preventing coronary artery lesions [10]. In this study, we included patients aged six months to five years, and the duration of fever and the acute phase of the disease was relatively similar between the two groups, so we had no additional factor in increasing the risk of coronary artery involvement.

Corticosteroids have been considered contraindicated in KD because of the higher prevalence of coronary aneurysms in the corticosteroid-treated group than in the untreated KD group due to the results obtained from the Kato study. However, the results of this study can not be relied upon due to the inappropriate method of study and administration of corticosteroids, including low-dose medication, oral administration, and the timing of steroid treatment too late [25].

The type I interferon pathway is affected in patients treated with pulse corticosteroids but not oral corticosteroids. Pulse corticosteroids markedly but transiently decreased the number of plasmacytoid dendritic cells. Intravenous pulse therapy can normalize the interferon (IFN) signature. This finding correlates with a reduction in plasmacytoid dendritic cells (PDCs) but not other cells, such as CD141 monocytes, in the blood [26].

There are also some studies on the use of corticosteroids in the treatment of KD alone. According to a study by Kijima et al., intravenous methylprednisolone pulse could significantly reduce coronary artery damage in children with KD. In this study, 60 patients with KD diagnostic criteria were treated with intravenous methylprednisolone pulse at a 30 mg/kg dose for three consecutive days, and heparin was performed. Coronary artery disease was significantly reduced in the pulse therapy group [27].

Shinohara et al. also showed a possible role of corticosteroids in treating the acute phase of KD [22]. Singhal et al. described the course of a one-year-old child with KD treated with aspirin and corticosteroids as the initial therapy. A follow-up showed the presence of a small-sized coronary aneurysm [28].

Pulsed administration regimens have renewed interest in corticosteroids, particularly high doses of 250-1000 mg methylprednisolone for one to five days. It was reported to be well-tolerated, more immunomodulatory than immunosuppressive, and safe, with only minor dose-related side effects including flushing, mild hypertension, gastritis, weight gain, depression, hyperglycemia, insomnia, palpitations, sinusitis, and urinary tract infections [29,30].

Even though, in our study, the frequency of coronary artery involvement was not significantly different from before treatment between the groups, patients in the IVMP group showed marked improvement in coronary artery involvement after two weeks in comparison before treatment. TNF-α in the acute phase of KD and interleukin 6 in the acute and sub-acute phases of KD are higher than in the recovery phase, which can justify the greater prevalence of coronary artery abnormalities at this stage [20]. Given this critical point and cytokine changes, the ability of steroid pulses to reduce coronary artery involvement in the sub-acute phase also appears to be related to controlling the levels of cytokines involved in the subacute phase, as our study shows this effect.

Based on the available evidence, this lack of efficacy of corticosteroids in first-line combination therapy in some patients may be due to reduced corticosteroid receptor gene expression on mRNA in these populations [16]. The basic idea of our pulse therapy was to prevent or suppress the process of aneurysm development by treating coronary angiitis, which may underlie coronary changes in KD, using large doses of corticosteroids parenterally within a relatively short period in the early stage of the disease. The present study indicates that steroid pulse therapy is effective in patients with some dilating coronary artery changes and patients with an aneurysm formation. Theoretically, such a treatment should be initiated before dilative lesions appear in the acute phase of KD. In the acute phase, the activation of the innate immune system is demonstrated cytokine storm. In IVIG-resistance KD patients and patients with coronary artery involvements, TNF-α and IL6 are higher than in IVIG-responder patients [20,31]. Methylprednisolone pulses, especially in suppressing the innate immune system during the acute phase of KD, may have a more pivotal role in inhibiting progressive coronary involvements than IVIG as the first-line treatment.

It should be emphasized that we found no significant side effects in any patient and that there was no convincing evidence that steroids made the coronary lesions worse. In a systematic review of eight studies with 1877 patients, none reported long-term coronary morbidity after one year [24]. Recent studies on Kawasaki-like disease following COVID infection showed IVMP alone or in combination with IVIG had a lower risk for cardiovascular involvement than IVIG alone [32-34].

Therefore, at present, intense corticosteroid pulse therapy for the coronary artery's inflammatory process may be used to prevent or minimize any tragic sequel of KD. Further evaluation with a larger sample size might be more valuable for IVIG and IVMP comparisons as the first-line treatment in KD, especially in anticipation of IVIG-resistance patients.

Our study is the first on IVMP pulse therapy first-line treatment of KD alone as a clinical trial study. However, there are some limitations to our study. A single-blinded study and two-month follow-up are the main limitations of this study. A large multi-center study with a diverse population demographic is required to influence a significant change in the current management guidelines for KD. Another diagnostic limitation was the impossibility of measuring cytokine levels at different stages of the disease and comparing them in two groups to prove the effect of steroid pulses on cytokine levels. Because there are no criteria for diagnosis of atypical Kawasaki, we had to exclude atypical Kawasaki from this study. However, incomplete KD, based on AHA criteria, was included.

## Conclusions

IVMP can effectively control systemic inflammation in KD. According to our study, it may reduce coronary artery damage in children with KD like IVIG. Like other vasculitis, IVMP may be an appropriate alternative treatment for KD, where IVIG may not be readily available. Based on this study, if a definitive benefit or equivalence between initial aspirin plus corticosteroid and the standard regimen can be conclusively demonstrated in large randomized control trials (RCTs), the use of corticosteroids may be justified.
